# Supplementary material for: From sequence to enzyme mechanism using multi-label machine learning
Source: BMC Bioinformatics. 2014 May 19;15:150. doi: 10.1186/1471-2105-15-150 (PMC4229970; doi:10.1186/1471-2105-15-150)
Supplement: Additional file 2 — Java code of ml2db. Additional file ml2db_code.tar.gz contains the Java source code to run the multi-label machine learning experiments and save the results to database. The code’s Javadoc is included. [file 1471-2105-15-150-S2.zip › additional file 2/ml2db/utils/doc/uk/ac/standrews/utils/main/stats/class-use/TruncatedPareto.html]

Uses of Class uk.ac.standrews.utils.main.stats.TruncatedPareto


JavaScript is disabled on your browser.


- Overview
- Package
- Class
- Use
- Tree
- Deprecated
- Index
- Help

- Prev
- Next

- Frames
- No Frames

- All Classes

## Uses of Class uk.ac.standrews.utils.main.stats.TruncatedPareto

- Packages that use TruncatedPareto

  | Package | Description |
  | --- | --- |
  |  |  |
  | --- | --- |
  | uk.ac.standrews.utils.main.stats |  |
  | uk.ac.standrews.utils.test.stats |  |
- - ### Uses of TruncatedPareto in uk.ac.standrews.utils.main.stats

    Subclasses of TruncatedPareto in uk.ac.standrews.utils.main.stats

    | Modifier and Type | Class and Description |
    | --- | --- |
    |  |  |
    | --- | --- |
    | `class` | `PseudoTruncatedPareto` A truncated pareto distribution which stores a big lists of power-law extracted random numbers. |
  - ### Uses of TruncatedPareto in uk.ac.standrews.utils.test.stats

    Methods in uk.ac.standrews.utils.test.stats that return TruncatedPareto

    | Modifier and Type | Method and Description |
    | --- | --- |
    |  |  |
    | --- | --- |
    | `static TruncatedPareto` | TruncatedParetoTest.`getTruncatedPareto100()` |

- Overview
- Package
- Class
- Use
- Tree
- Deprecated
- Index
- Help

- Prev
- Next

- Frames
- No Frames

- All Classes
